# A Rare Case of Pyogenic Granuloma in the Tooth Extraction Socket

**DOI:** 10.1155/2021/5575896

**Published:** 2021-05-21

**Authors:** Yosuke Iijima, Nami Nakayama, Leona Kashimata, Miki Yamada, Ryutaro Kawano, Shunsuke Hino, Takahiro Kaneko, Norio Horie

**Affiliations:** ^1^Department of Oral and Maxillofacial Surgery, Saitama Medical Center, Saitama Medical University, Saitama, Japan; ^2^Department of Pathology, Saitama Medical Center, Saitama Medical University, Saitama, Japan

## Abstract

Pyogenic granuloma (PG) refers to a common, acquired, benign, and vascular tumor that arises in tissues such as the skin and mucous membranes. However, it is extremely rare for PG to arise from an empty socket after tooth extraction. Herein, we describe a rare case of PG that arose from the empty extraction socket of the second molar adjacent to a dentigerous cyst of the left mandibular wisdom tooth in a 57-year-old man. The patient's second molar was extracted during the same procedure in which the wisdom tooth and cyst were removed. Subsequently, at 42 days after surgery, an exophytic mass was found in the socket of the second molar. An excisional biopsy was performed, and the histopathological diagnosis was PG. Since the PG recurred 90 days after the surgery, a complete excision with bone curettage was performed. During the 12 months of follow-up, there has been no recurrence observed. In conclusion, as tooth extraction is a routine dental practice, clinicians should be aware that PGs can also develop from an extraction socket.

## 1. Introduction

Pyogenic granuloma (PG) refers to a common, acquired, benign, and vascular tumor that arises in tissues such as the skin and mucous membranes [1]. Although PGs were previously thought to be a reactive exaggerated granulomatous product, a recent classification by the International Society for the Study of Vascular Anomalies has classified PG, which is also known as lobular capillary hemangioma, as a benign vascular tumor [2]. In addition, epulis gravidarum, which appears in the gingiva of pregnant women and is well known to both dentists and oral surgeons, has also been included in this new classification of PG. With regard to the etiology of the development of PGs, it has been speculated that there is an association with female sex steroid hormones, in addition to certain kinds of drugs and viruses [3]. However, there are many cases where the etiology remains unclear.

The oral mucosa is a common site for PG, with the most frequent location found to be the gingiva followed by the lower lip and tongue [3]. Oral PG exhibits a solitary, red, sessile, or pedunculated papule that is very friable. The tumor easily bleeds and may be accompanied by surface ulceration [3]. Although PG is usually slow-growing, at times, it can show rapid growth, with large PG becoming lobulated [1, 4]. Histologically, PG consists of highly vascular granulation tissue with a variegated inflammatory infiltrate, but, with maturation, fibrosis is strongly observed [5].

Although the oral mucosa is a common site for PG, it is extremely rare for PG to arise from an extraction socket [6, 7]. In this case report, we present a rare case of PG that arose from a tooth extraction socket of the tooth that was adjacent to a dentigerous cyst, which was extracted at the same time as the removal of the cyst.

## 2. Case Report

**Figure 1:**
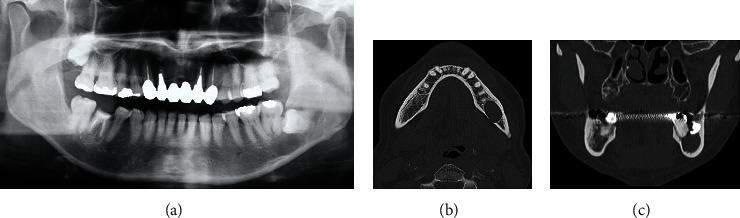
Photoradiograph of the impaction of the left mandibular wisdom tooth and surrounding cystic radiolucent image: (a) orthopantomogram; (b) computed tomography of axial image; (c) computed tomography of sagittal image.

**Figure 2:**
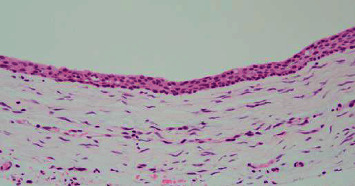
Photomicrograph of the dentigerous cyst. The cystic wall, which is covered by flat squamous cell epithelium, is composed of fibrous connective tissue with scarce inflammatory infiltrate (H-E staining, original magnification ×20).

**Figure 3:**
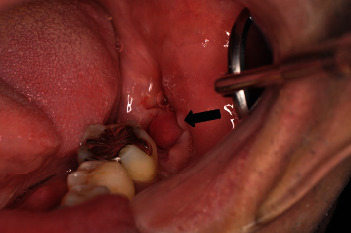
Reddish granular tissue that easily bleeds is observed protruding from the mesial wall of the extraction socket of the second mandible (arrow).

**Figure 4:**
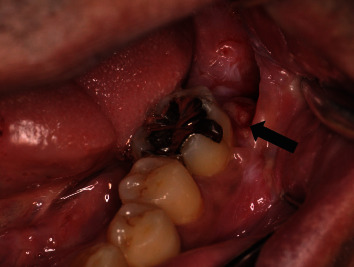
The excised site after one week. A stump of the mass can be seen on the mesial wall of the extraction socket (arrow).

**Figure 5:**
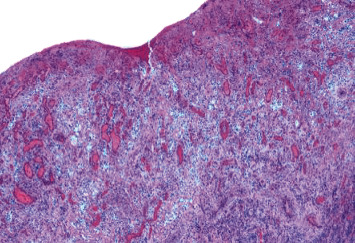
Photomicrograph of the mass in the tooth extraction socket. The specimen is partially covered with acanthotic squamous cell epithelium that is composed of granulation tissue with prominent small capillaries and large amounts of inflammatory infiltrate such as lymphocytes, plasma cells, and leukocytes. The endothelial cells often have plump cytoplasm (H-E staining, original magnification ×40).

**Figure 6:**
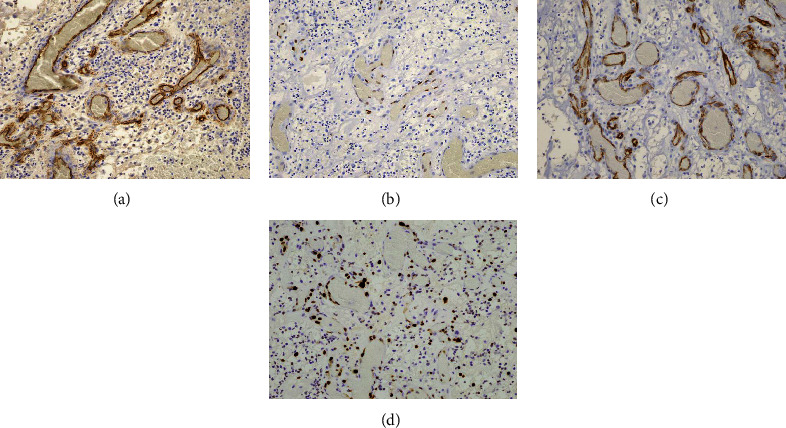
Immunohistochemical staining. The endothelial cells are positive for CD34 (a), ERG (b), and WT-1 (c) (each original magnification ×100). Approximately 5% of the endothelial cells are positive for Ki-67 (d) (original magnification ×100).

A 57-year-old male was referred to our oral surgery clinic for the evaluation of a radiolucent image of his left mandible. Three weeks prior to being seen in our clinic, he visited a family dentist for the treatment of caries in his right maxillary tooth. At that time, a relatively large left mandibular radiolucent area, which was larger than that previously observed, was found. The patient's past history included hypertension, hemiplegia, dysarthria, and dysphagia. At the time of his initial examination, he was taking amlodipine besilate 2.5 mg/day. Radiographic examination showed impaction of the left mandibular wisdom tooth and a cystic radiolucent area spreading around the crown of the tooth (Figure 1). Oral examination demonstrated that there was no gingival swelling or tenderness in the area corresponding to the cyst and wisdom teeth. The patient's oral hygiene and periodontal status were fair. The clinical diagnosis was a dentigerous cyst of the left mandibular wisdom tooth. As results suggested that further expansion of the cyst was possible, after the consultation with the attending physician, the patient, and his family, a surgical treatment was planned. Under general anesthesia, the wisdom tooth and cyst were removed. The surgical procedure was performed using a triangular flap. This involved an incision from the distobuccal edge of the left first molar, dropping at a slightly oblique angle into the mandibular vestibule. The second incision made was a relieving incision that ran from the ramus to the distobuccal aspect of the first molar [8]. The left second molar was extracted at the same time, as the root apex was contained in the cyst. No abnormal findings were observed in the excision cavity of the cyst, the extracted mandibular second molar, or their extraction sockets. The surgical wound was sutured without complication. The histopathological diagnosis of the specimen was a dentigerous cyst (Figure 2). A follow-up was performed at approximately 1, 3, and 6 weeks after surgery. Around the time of his third follow-up, which was 42 days after his surgery, rapid proliferation of granulomatous tissue was found in the extraction socket of the second molar (Figure 3). However, the patient was not aware of when the mass first initially developed. The examination showed that the mass was slightly reddish, easily bled, was 13 × 8 mm in size, and was pedunculated from the mesial wall of the extraction socket of the second molar. There was no association found between the granulomatous tissue and the wound of the cyst. Due to a concern regarding the possibility of a malignant tumor, an excisional biopsy was performed.  Figure 4 shows the excision site one week after the biopsy. A stump of the original mass was seen on the mesial wall of the extraction socket. Histopathologically, the specimen was partially covered by acanthotic squamous cell epithelium with ulceration and composed of granulation tissue with prominent capillary-sized vessels. In addition, there were large amounts of inflammatory infiltrate such as lymphocytes, plasma cells, and leukocytes present. The endothelial cells often had plump cytoplasm with a thick wall (Figure 5). Immunohistochemically, CD34, erythroblast transformation-specific-related gene (ERG), and WT-1 were positive for endothelial cells, with Ki-67 positivity found in approximately 5% of the epithelial cells (Figure 6). The histopathological diagnosis was PG. Although the growth of granulation tissue temporarily subsided, recurrence of the granulation tissue was found on the day of the follow-up, which corresponded to the 90th day after the surgery. As a result, we performed a complete removal with bone curettage, while the patient was under local anesthesia. Histopathological findings of the second specimen were similar to that for the first specimen, with the same diagnosis of PG. During the subsequent 12 months of follow-up, there has been no recurrence observed.

## 3. Discussion

Descriptions of PG in the extraction socket are extremely rare. Only 4 cases including our current case have been previously reported [6, 7]. Including our current case, all 4 of these cases exhibited a similar clinical presentation with an asymptomatic granulation tissue-like exophytic mass in the extraction socket. It has also been reported that this surface can be ulcerated due to the stimulation caused by occlusion and mastication. The period from tooth extraction to onset was reported to range from 1 to 2 months [6].

Histologically, PG consists of lobular aggregates of capillary-sized vessels, which are lined by plump endothelial cells, with scattered fibroblasts and various amounts of inflammatory infiltrate. Immunohistochemically, although these endothelial cells were positive for vascular markers such as CD31, CD34, factor VIII antigen, and WT-1, they were negative for glucose transporter protein isoform-1 (GLUT-1) [9, 10]. WT-1 is a tumor suppressor gene that plays an important role in regulating hematopoiesis and angiogenesis [9]. Ki-67, which is a proliferation marker, has been reported to exhibit a positive ratio for the Ki-67 labeling index of PG, ranging from 4 to 10% [9]. In the subjects with PG that arose from an empty socket, immunohistochemical staining was performed in one case, with results showing that CD31 was positive. Immunohistochemical staining in our current study also found that there was positive staining for CD34 and WT-1. In addition, ERG, which is essential in endothelial differentiation and angiogenesis, also showed positive staining [11]. The positivity of the Ki-67 labeling index in our current study was approximately 5%.

Etiologically, a history of trauma, hormonal factors, infection, and certain medications have all been suggested to be causes of PG [12–14]. Furthermore, BRAF mutations and potentially herpes virus type 1, Orf virus, and/or human papillomavirus type 2 have also been recently recognized to be an important part of the pathogenesis of PG [1, 15–19].

Although the oral mucosa can easily be affected by trauma, a history of trauma has only been reported in oral lesions in approximately 7% of all cases [9]. In our current case, the occurrence was thought to be related to a concomitant dentigerous cyst, as it occurred after the removal of this cyst. However, our findings strongly suggested that the PG was derived from the adjacent tooth extraction socket, as the base of the pedunculated PG was located on the mesial surface of the second molar extraction socket, which was a long distance from the cyst wound. Although this case occurred in the empty tooth extraction socket and it is known that the periodontal ligament disintegrates and disappears after tooth removal, our findings suggested that it was derived from the adjacent gingival cells rather than the periodontal ligament cells. Regardless, the findings for this case suggested an association with either tooth extraction or trauma. Although tooth extraction is a clinically common practice, PGs originating from the extraction socket are very rarely observed during these procedures. The reason for this could possibly be that there are additional cofactors other than trauma. However, the precise reason remains unknown at the present time.

With regard to the effect of hormones on oral lesions, such as epulis gravidarum, it has been suggested that estrogens and other sex hormones can exaggerate the inflammatory responses in gingival tissue, particularly during pregnancy [20]. Medications that may cause PG include anticancer agents such as pyrimidine analogs, taxanes, epidermal growth factor receptor inhibitors, tyrosine kinase inhibitor and BRAF inhibitors, and immunosuppressants such as TNF-alpha antagonists and mTOR inhibitors [1, 21]. In these types of drug-associated cases, it has been reported that PG can occur in multiple sites. Therefore, care must be taken with regard to these drugs, as they have often been used for cancer chemotherapy in recent years.

Bacterial infection has additionally been considered to be a factor that can cause PG. Moreover, it has been suggested that periodontal disease was a factor associated with PG in one PG case that was related to an empty tooth extraction socket. Even so, it has been recently denied that the effects of a bacterial infection are associated with the pathology of PG, as there have yet to be any bacterial strains definitively identified [22]. However, it should be noted that the oral hygiene practice and periodontal status of our patient were fair and not exceedingly poor, respectively.

Normally, it is uncommon for PGs to spontaneously regress. With regard to the treatment of oral PGs, surgical intervention is the preferred method. However, since PGs can sometimes reoccur, it has been reported that a complete excision of the entire lesion results in the lowest rate of recurrence. In addition, laser treatment may also be useful [6]. Even so, the effectiveness of using a conservative medication therapy has yet to be established. Furthermore, needless to say, there is a chance that epulis gravidarum may regress after the delivery [23].

The differential diagnosis of PG in the oral region includes the presence of a disease showing an exophytic mass-like growth. Thus, malignant tumors initially need to be considered. Moreover, malignant tumors include not only primary cancer but also hematological and metastatic cancers [24]. Benign tumors and inflammatory disease include findings such as peripheral fibroma and giant cell lesions [4]. In the case of maxillary tooth extraction, herniation of a sinonasal polyp is also possible [25]. Thus, a patient's medical history, radiographic examination, and blood tests can all be of assistance in the diagnosis. However, a histopathological search is essential for making a definitive diagnosis. In our current case, the histopathological search also proved to be very effective.

In conclusion, since tooth extractions are a routine dental practice, clinicians need to be aware that PGs can additionally develop from an extraction socket.
